# Seasonal variation in structure and function of gut microbiota in *Pomacea canaliculata*

**DOI:** 10.1002/ece3.9162

**Published:** 2022-07-29

**Authors:** Shuxian Li, Zijin Qian, Jiani Yang, Youfu Lin, Hong Li, Lian Chen

**Affiliations:** ^1^ Jiangsu Key Laboratory for Biodiversity and Biotechnology College of Life Sciences, Nanjing Normal University Nanjing China; ^2^ College of Biology and the Environment, Nanjing Forestry University Nanjing China

**Keywords:** 16S rRNA, microbiome, *Pomacea canaliculata*, seasonal variation

## Abstract

Gut microbiota is associated with host health and its environmental adaption, influenced by seasonal variation. *Pomacea canaliculata* is one of the world's 100 worst invasive alien species. Here, we used high‐throughput sequencing of the 16S rRNA gene to analyze the seasonal variation of gut microbiota of *P. canaliculata*. The results suggested that the predominant gut microbial phyla of *P. canaliculata* included Firmicutes and Proteobacteria, which helped digest plant food and accumulate energy. The gut microbiota of *P. canaliculata* in summer group showed the highest diversity, whereas the winter group possessed the lowest, probably due to the shortage of food resources of *P. canaliculata* in winter. Principal coordinate analysis analysis based on unweighted unifrac and weighted unifrac indicated that the composition of gut microbiota of *P. canaliculata* significantly varied across seasons. Bacteroidetes tended to be enriched in summer by linear discriminant analysis effect size analysis. Actinobacteria and Cyanobacteria were extremely abundant in autumn, while Fusobacteria and *Cetobacterium* enriched in winter. In conclusion, the structure of the gut microbiota of *P. canaliculata* was significantly different among seasons, which was beneficial to the environment adaptation and the digestion and metabolism of food during different periods.

## Graphical abstract

We collected 28 gut content samples of Pomacea canaliculata from Suzhou city over three seasons and analyzed the seasonal variation of gut microbiota using 16S rRNA gene V3‐V4 sequencing.The gut microbiota of summer group showed the highest diversity, while the winter group showed the lowest.The structure of the gut microbiota of P. canaliculata were significantly different among seasons, which was beneficial to the environment adaptation and the digestion and metabolism of food during different periods.

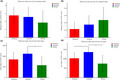

## 1 INTRODUCTION

The intestinal microbiome represents the collective interacting genomes and symbiotic microorganisms in the intestinal tract (Kinross et al., 2011). The gut microbiota is complex and dynamic microbial ecosystem (Johnson et al., 2019), being sensitive to perturbations, such as dietary changes, environmental factors, and enteric pathogens (Ren et al., 2017), which has co‐evolved with hosts and play an integral role in nutrient intake, behavior, metabolism, immune function, and development of the host (Heijtz et al., 2011; Sonnenburg & Bäckhed, 2016). Gut microbiota may influence host evolution by expanding host dietary niches (Alberdi et al., 2016; Moran et al., 2019), influencing the evolution of host phenotypic plasticity in response to environmental change and development (Gilbert et al., 2015), and generating selection on hosts for traits that benefit host fitness (Foster et al., 2017).

Changes in food resources and seasonal fluctuations in ambient temperature alter microbial communities (Maurice et al., 2015; Moschen et al., 2012; Turnbaugh et al., 2009). The gut microbiota of animals is indirectly affected by environmental temperatures and directly influenced by hosts' physiological responses to seasonal changes in food sources (Amato et al., 2015; Solden et al., 2017; Stevenson et al., 2014). Tong et al. (2020) found that the composition and structure of skin and gut microbiotas of *Rana dybowskii* changed between summer and winter due to activity levels, environmental conditions, nutritional, and immune status. The microbiota composition of digestive glands of *Mytilus galloprovincialis* was significantly different along with collection seasons affected by the environmental conditions in sampling area (Wathsala et al., 2021).

*Pomacea canaliculata* is a freshwater snail native in South America and is listed as one of the 100 world's invasive alien species by the International Union for Conservative of Nature and the Invasive Species Specialist Group (Lowe et al., 2000). After the 1980s, *P. canaliculata* was introduced to many countries in North America, Europe, East and Southeast Asia as aquarium pets or human food (Hayes et al., 2008). Dietary of *P. canaliculata* was flexible, not only consuming crops and aquatic macrophytes but also preying on small snails and other aquatic animals (Carlsson et al., 2004; Kwong et al., 2009). *P. canaliculata* also has high fecundity, fast growth, together with a lack of effective natural enemies in invaded wetlands, which are highly adaptable to harsh environmental conditions, such as low dissolved oxygen concentration, high nutrient content, low food supply, and temperature (Cowie, 2002). In temperate winter, *P. canaliculata* hibernated through burying themselves into the topsoil, slowing down metabolism and entering dormancy in paddy fields, irrigation canals, ponds and other bodies of water to possess the acquisition of cold hardiness (Matsukura et al., 2008, 2009; Zhou et al., 2003). *P. canaliculata* have become serious agricultural and ecological pests, causing massive economic losses (Cowie, 2002). *P. canaliculata* have established natural populations at least 11 provinces in southern China (Yang et al., 2018) were listed as one of the first national key invasive alien species under management in China.

Previous studies of biological characteristics of *P. canaliculata* in different seasons have mainly focused on the host reproduction, growth, and temperature adaptation (Matsukura et al., 2008; Seuffert & Martin, 2017). The number of eggs per egg mass decreased as food availability reduced in winter to increase hatchling survival. Compared with winter, the hatchling survival of *P. canaliculata* in summer was higher, mainly due to the influence of the ambient temperature (Tamburi & Martín, 2011). The cold treatment test of *P. canaliculata* showed that the cold resistance was increasing along with the decrease in temperature and the increase of habitat displacement. *P. canaliculata* were treated at 0°C for 5 days on December, with a result of almost all *P. canaliculata* surviving (Matsukura et al., 2016). The cold tolerance of *P. canaliculata* collected from paddy fields in summer was significantly enhanced (Wada & Matsukura, 2007).

With the development of high‐throughput sequencing, several studies have explored the intestinal microbiota of *P. canaliculata* using theV3‐V4 regions of the 16S rRNA gene. Li et al. (2019) studied the diversity and composition of the microbiota of the buccal masses, stomachs, and intestines of *P. canaliculata*. The diversity of the microbiota was highest in the intestine but lowest in the buccal mass. The composition of the microbiota was diverse among the different gut sections. Significant differences were found in the structure of gut microbiota among female, male, and juvenile groups, suggesting the gut microbiota of *P. canaliculata* has been affected by the developmental stages (Chen et al., 2021). Zhou et al. (2022) investigated difference of gut microbiota between *P. canaliculata* and native snail *Cipangopaludina chinensis*. The results found that there were marked differences in the gut microbiota structure between *P. canaliculata* and *C. chinensis*. Unique or high microbial taxa were more abundant in *P. canaliculata*, indicating that this invasive snail has an enhanced potential to adapt to new habitats. Most studies have explored the gut microbiota of *P. canaliculata* affected by gut sections, sex, the developmental stages. However, the influence of environmental condition such as seasonal variation on the gut microbiota of *P. canaliculata* has been limited explored. Studying the influence of seasonal variation on the gut microbiota of *P. canaliculata* allows us to figure out whether microbial variations of *P. canaliculata* in different seasons enables the host in response to different environmental conditions.

In the present study, we aimed to explore the differences in the gut microbiota of *P. canaliculata* among seasons. Information concerning the influence of seasonality on the *P. canaliculata* gut microbiome may help to understand how the gut microbiome is affected by different seasons. Studying the importance of seasonal variation in reshaping the gut microbiota of *P. canaliculata* can help understanding the relationship between microbiota and environmental adaption in this invasive snail.

## 2 MATERIALS AND METHODS

### 2.1 Sample collection

A total of twenty‐eight *P. canaliculata* were collected from a pond in Suzhou City (31.46°N, 120.95°E), Jiangsu Province, China, from July 2020 to January 2022, including the summer group (with 6 females and 5 males), the autumn group (with 5 females and 5 males), and the winter group (with 4 females and 3 males) (Table 1). The water temperatures of the sampling sites were recorded among seasons during sampling in the field. The water temperatures in July, November, and January were 32.60°C, 18.58°C, 7.79°C, respectively. *P. canaliculata* in winter were generally sampled under the soil by fishing net because snails overwinter were buried in the bottom sediments. All experimental individuals were wiped by 75% ethanol three times, followed by rinsing twice in distilled water to sanitize the surface prior to dissection and removing the shell from each snail (Chen et al., 2021). Coiled gut contents were extracted carefully to avoid rupturing the gut wall. Each sample was stored in sterile tubes using liquid nitrogen and later stored in a freezer of −80°C.

**TABLE 1:** Sample information of different seasons

Season	Sampling date	Number of females	Number of males	Shell height (mm)
Summer	July 2020	6	5	42.6200 ± 1.9700
Autumn	November 2020	5	5	39.8000 ± 1.4900
Winter	January 2022	4	3	50.0400 ± 2.9100

### 2.2 DNA extraction and sequencing

Microbial DNA was extracted from the intestinal samples using FastDNA® Kit (MP Biomedicals, CA, USA) according to the instructions of the manufacturer. DNA quantity was examined by 1% agarose gel electrophoresis. The universal primers 338F(5′‐ACTCCTACGGGAGGCAGCAG‐3′) and 806R (5′‐GGACTACHVGGG TWTCTAAT‐3′) were used to amplify the V3–V4 region of 16S rRNA gene (Lin et al., 2019). PCR products were purified and subjected to high‐throughput sequencing using the Illumina MiSeq PE300 platform by Majorbio BioPharm Technology Co., Ltd (Shanghai, China).

### 2.3 Statistical and bioinformatic analyses

Raw data were processed using the QIIME software (version 1.9.1, http://qiime.org/install/index.html). Raw fastq files were quality filtered by Trimmomatic and merged by FLASH with the criteria as previously described (Chen et al., 2021). Sequences were assigned to the operational taxonomic units (OTUs) with a 97% identity threshold by USEARCH (version 7.1, http://drive5.com/uparse/) (Edgar et al., 2011). The Ribosomal Database Project (RDP) Classifier (http://rdp.cme.msu.edu/) was employed for taxonomy assignment of each 16S rRNA gene sequence against Silva 16S rRNA database (Version 132, http://www.arb‐silva.de) (Quast et al., 2013). Taxonomic identity of the noranked OTUs (mean relative abundance >1%) on genus level was queried using BLAST against the NCBI database.

Rarefaction curves were plotted for each sample to determine the abundance of communities and sequencing data of each sample. For the alpha‐diversity metrics, Ace, Chao1, Shannon, Simpson indices were calculated using QIIME and the Kruskal‐Wallis *H* tests. Principal coordinate analysis (PCoA) was used based on the unweighted and weighted UniFrac distances and analysis of similarity (ANOSIM) based on 999 permutations (Clarke, 1993; Warton et al., 2012). A Venn diagram was generated to describe unique and common OTUs among different seasons using R software (version 3.1.0, R Core Team, Auckland, New Zealand). Linear discriminant analysis effect size (LEfSe) was used to analyze the differences in intestinal microbial composition among groups (|LDA score| > 3.5) (Segata et al., 2011). To identify statistically significant differences of intestine microbiota at the phylum and genus level among the groups, Kruskal‐Wallis *H* test was used by SPSS 19.0 software. *p*‐value <.05 was considered statistically significant.

PICRUSt (Langille et al., 2013) was used to predict microbial functions based on the Kyoto Encyclopedia of Genes and Genomes (KEGG) and Evolutionary Genealogy of Genes: Non‐supervised Orthologous Groups (EggNOG) databases. LEfSe was applied to analyze differences in the functionality of the gut microbial community among the three groups (|LDA| > 3.5).

## 3 RESULTS

### 3.1 Sequencing depth and alpha diversity indices

DNA extracted from twenty‐eight *P. canaliculata* samples was amplified successfully, and 556,080 valid sequences were obtained. *P. canaliculata* yielded 3085 valid OTUs at a 97% identity. OTUs were assigned into 44 phyla, 121 classes, 274 orders, 472 families, and 972 genera. The rarefaction curves for all the samples showed the observed species number gradually stabilized (Figure S1), indicating that the sequencing data were reasonable and that there was uniform species composition within the sample. These results indicated that the sample size in this study was sufficient for follow‐up analysis.

**FIGURE 1:**
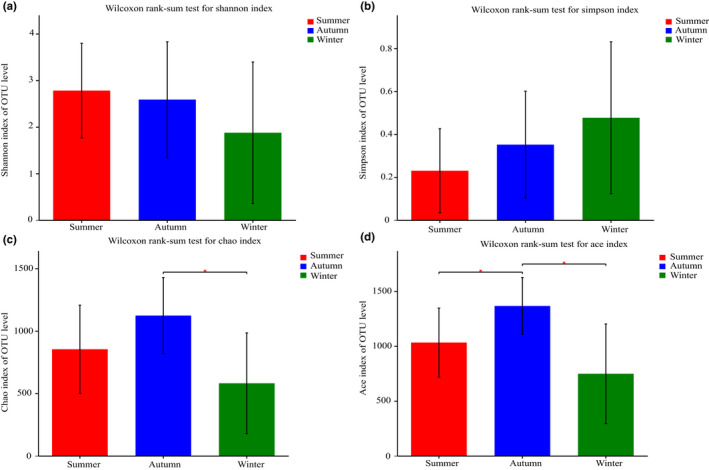
Comparison of alpha diversity index of intestine microbiome in different seasons of *Pomacea canaliculate*. (a): Shannon index; (b): Simpson index; (c): Chao1 index; (d): Ace index. Significant differences were marked as “*” (*p* < .05) and “**” (*p* < .01).

The Shannon index in the summer group was higher than that in the autumn group, and the Shannon index in the autumn group was higher than that in the winter group, while the Simpson index was opposite (Table S1). However, there was no significant difference in Shannon and Simpson index among these three groups (*p* > .05). The intestinal microbial diversity of *P. canaliculata* was the highest in summer and the lowest in winter. There was no significant difference among three groups (*p* > .05; Figure 1a,b).

### 3.2 Taxonomic composition and beta diversity analysis

**FIGURE 2:**
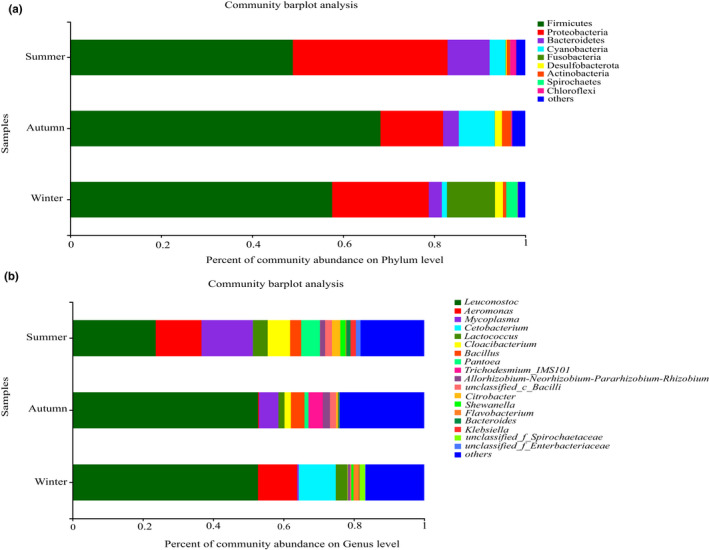
Composition of the bacterial community in the guts of *Pomacea canaliculata* in different seasons (mean relative abundance>1%). (a): At the phylum level; (b): at the genus level.

The predominant phyla of the intestinal microbiome of *P. canaliculata* in summer, autumn and winter were similar, but the proportions of each phylum were different (Figure 2a). The dominant bacterial phyla detected within gut microbiota of *P. canaliculata* in three groups were Firmicutes and Proteobacteria (Figure 2a). Except for these two phyla, the relative abundance of bacterial phyla in different seasons was variable. Microbiota of the summer group were also enriched with two phyla, including Bacteroidetes (9.25%), and Cyanobacteria (3.49%), while microbiota of the autumn group were enriched in the phyla Cyanobacteria (7.87%) and Bacteroidetes (3.46%), and the winter group were enriched with the Fusobacteria (10.57%), Bacteroidetes (2.87%), and Spirochaetes (2.51%).

The intestinal flora of *P. canaliculata* in different seasons varied greatly at the genus level (Figure 2b). The most abundant bacterial genus across all samples was *Leuconostoc*, accounting for 23.66%, 52.78%, 52.75% in summer, autumn, and winter, respectively. In summer, the relative abundances in descending order were *Mycoplasma* (14.59%), *Aeromonas* (13.02%), *Cloacibacterium*, (6.40%), *Pantoea* (5.35%), *Lactococcus* (4.21%), and *Bacillus* (3.14%). In autumn, the relative abundances are in descending order of *Mycoplasma* (5.53%), *Trichodesmium_IMS101*, (4.09%), and *Bacillus* (3.85%). In winter, the dominated genera were *Aeromonas* (11.12%), *Cetobacterium* (10.52%), and *Lactococcus* (3.28%).

**FIGURE 3:**
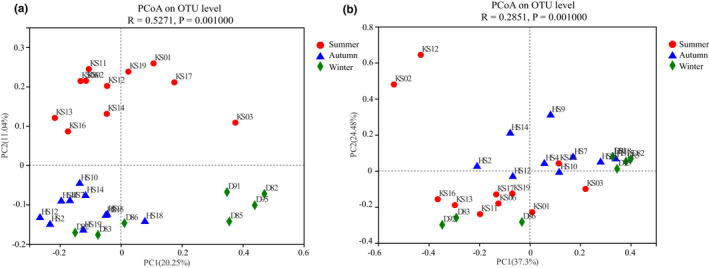
PCoA analysis based on (a): unweighted and (b): weighted Unifrac distances of intestine microbiome from different seasons.

The PCoA plot of unweighted UniFrac distance showed that the difference in the gut microbiota structure was not significant between female and male *P. canaliculata* among three groups (all *p* > .05). The composition of the intestinal microbiota community was significantly different in three groups (*R* = .5271, *p* = .001) (Figure 3a). This result was also supported by the weighted Unifrac‐based PCoA (*R* = .2851, *p* = .002) (Figure 3b).

### 3.3 Different intestinal microbiome of *P. canaliculata* in different seasons

**FIGURE 4:**
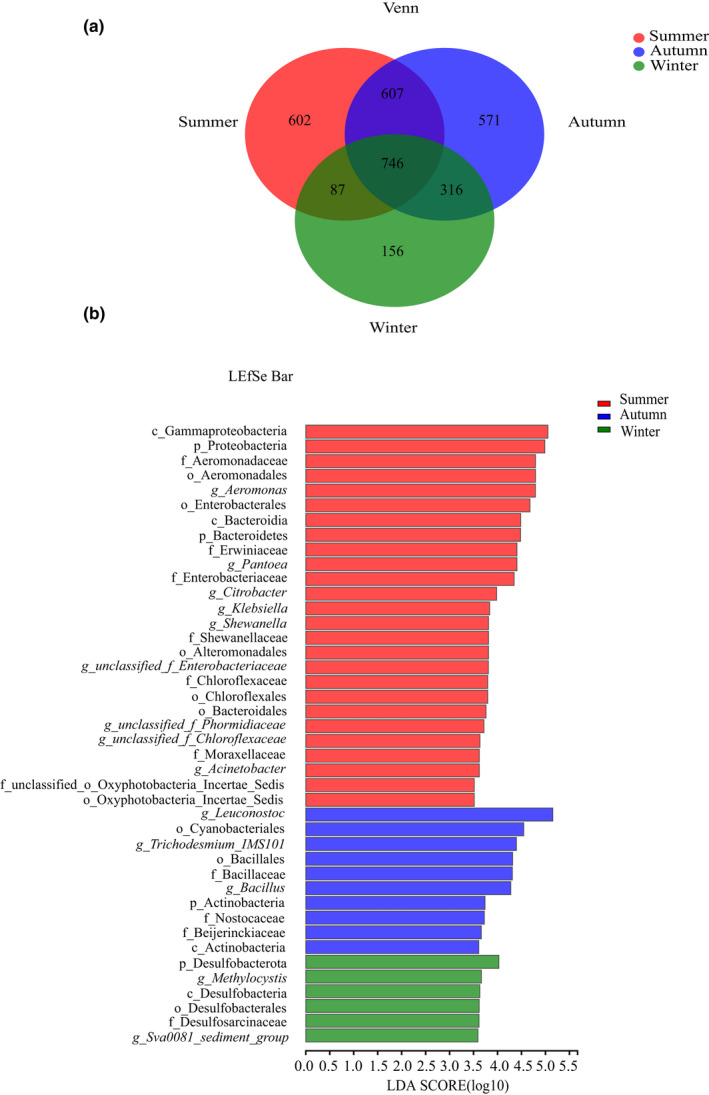
(a): Venn chart shows the common and unique OTU between the groups. (b): LEfSe analysis of *Pomacea canaliculata* intestine microbiome from different seasons (|LDA| > 3.5, *p* < .05). Histogram of the LDA scores computed for features differentially abundant between groups. The c, g, f, o, p in diagram represent class, genus, family, order, and phylum, respectively.

A total of 746 OTUs were shared in three groups, accounting for 36.53%, 33.30%, and 57.17% of the total number of the summer, autumn, and winter groups, respectively. There were 1296 unique OTUs in summer, 1494 unique OTUs in autumn, and 559 unique OTUs in winter (Figure 4a). Among these shared OTUs, 56.74% were from Firmicutes, 12.28% from Proteobacteria, 3.4% from Bacteroidetes, 2.89% from Fusobacteria, and 1.63% from Cyanobacteria.

LEfSe analysis was performed to identify species that differ significantly among groups (|LDA| > 3.5) (Figure 4b; Table S2). From the distribution histogram of LDA effect value, the intestinal microbiome of *P. canaliculata* in different seasons showed that Proteobacteria, Bacteroidetes, Gammaproteobacteria, Enterobacteriaceae, Chloroflexaceae, *Aeromonas*, *Shewanella*, *Acinetobacter*, *Citrobacter*, *Klebsiella* were significantly enriched in summer; Actinobacteria, Bacillaceae, Nostocaceae, and *Bacillus* were significantly enriched in autumn; Desulfobacterota, *Methylocystis* were significantly enriched in winter.

At the phylum level, the relative abundance of Cyanobacteria was the greatest in autumn, and the relative abundance of Fusobacteria was the greatest in winter based on the Kruskal‐Wallis *H* test (*H* = 14.6463, *p* = .0007) (Figure 6a; Table S3). At the genus level, the relative abundance of *Cetobacterium* was the greatest in winter (*H* = 17.5221, *p* = .0002), and the relative abundances of *Cloacibacterium* (*H* = 15.3263, *p* = .0005) and *Bacillus* (*H* = 15.4889, *p* = .0004) in summer and autumn was significantly greater than those in winter (Figure 6b; Table S4).

**FIGURE 5:**
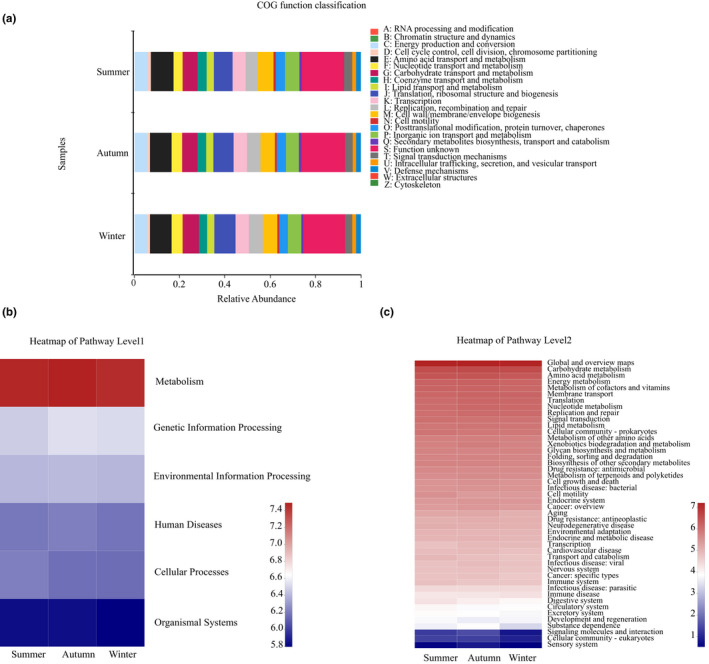
(a): Gut microbiota predict metabolic functions from EggNOG database in the summer, autumn, and winter group. Relative abundance column diagram of microbiota functions based on the KEGG database. (b): Microbiota functions are shown on the first level; (c): microbiota functions are shown on the second level.

A total of 23 metabolic functions were predicted in all samples from EggNOG database (Figure 5a). The principal functionality primarily consisted of amino acid transport and metabolism (9.67%), followed by translation, ribosomal structure and biogenesis (8.79%), carbohydrate transport and metabolism (6.87%), cell wall/membrane/envelope biogenesis (6.60%), etc. To better understand the functional differences, LEfSe analysis (|LDA| > 3.5) was conducted in the three groups (summer, autumn and winter). The results showed that no difference of function prediction was detected among these three groups.

A total of 7210 KEGG Orthology (KOs) were mapped to 404 level 3 KEGG pathways and were then classified into 46 level 2 KEGG pathways. At level 1, predicted functional pathways metabolism accounted for the highest proportion, followed by genetic information processing, environmental information processing, human diseases, cellular processes, organismal systems, etc (Figure 5b). At level 2, predicted functional pathways global and overview maps accounted for the highest proportion, followed by carbohydrate metabolism, amino acid metabolism, energy metabolism, and metabolism of cofactors and vitamins, etc (Figure 5c). LEfSe analysis identified that no difference of function prediction was detected among groups.

## 4 DISCUSSION

### 4.1 Structure and function of the intestinal microbes of *P. canaliculata* in different seasons

In this study, the alpha diversity of the gut microbiome was observed in different seasons. The highest diversity of gut microbiota was detected in summer group (Figure 1a,b). Previous studies suggested that higher α diversity leads to a more complex and stable intestinal microbiota composition, enhancing resistance to external interference and adaptability, which is beneficial to the host health (Stoffel et al., 2020). PCoA analysis based on unweighted and weighted UniFrac distance (Figure 3a,b) indicated that seasonal variations had significantly influence on the structure of gut bacterial community. Seasonal fluctuation is an important factor causing changes in the gut microbiota, which may be related to the environmental temperature (Pierce et al., 2016), habitat, and food composition (Baniel et al., 2021) and other factors. Temperature can induce significant changes in the gut microbiota and metabolism of European seabass (*Dicentrarchus labrax*) juveniles (Liu et al., 2022). Different habitat conditions in irradiance and riparian vegetation modulated the composition of the gut microbiota and their biochemical properties in the freshwater blackworms (*Lumbriculus variegatu*) (Kim et al., 2021). Furthermore, due to the gut microbiota of animals directly affected by hosts' physiological responses to seasonal changes in food resources, it responds to dietary fluctuations and presumably to adapt to new dietary niches (Amato et al., 2015). Microbiota variations of *Fejervarya limnocharis* in dominant gut microbiota at different seasons imply that frogs acquire different bacteria due to variations in their seasonal diet (Huang et al., 2021). Due to winter is a period of food shortage for most animals relative to summer and seasonal changes in the field foraging of *P. canaliculata*, intestinal microbiome gradually changes to adapt to this condition (Seuffert et al., 2010; Tamburi & Martín, 2011).

Although seasonal differences are generally considered to be the determinants of the seasonal changes of gut microbiota, a total of 33.30%–57.17% of OTUs were consistent in all three seasons. The common microbiota identified in shared OTUs were Firmicutes, Proteobacteria, Bacteroidetes, Fusobacteria, and Cyanobacteria. The common microbiota in different seasons plays an important role in maintaining the basic physiological function of the snail and the seasonal homeostasis of the gut microbiota (Hao et al., 2020).

The phyla Firmicutes and Proteobacteria were dominated in the gut of *P. canaliculata* among three groups (Figure 2a). The gut microbiota structure in the present study were similar to previous studies found in *P. canaliculata* (Chen et al., 2021) and *Crassostrea gasar* (Conceição et al., 2021), *C. gigas*, *C. sikamea*, and *C. corteziensis* (Fernández et al., 2014). Higher proportions of these two bacteria were often associated with diets containing plant ingredients (Rimoldi et al., 2018). *P. canaliculata* as an omnivorous species, except for the feed of small snails and other aquatic animals (Kwong et al., 2009; Kwong et al., 2010), they prefer to herbivorous food such as crops, phytoplankton, aquatic macrophytes (Carlsson et al., 2004). Firmicutes play an important role in the degradation of cellulose, helped to digest broadly herbivorous food. Firmicutes also have been reported to be able to promote preservation of gut homeostasis and host immunity development (Ben David et al., 2015). *Leuconostoc* belonged to the phylum Firmicutes was the most abundant genus in the gut of *P. canaliculata* (Figure 2b), which was also enriched in the autumn by LEfSe analysis (Figure 4b). *Leuconostoc*, which originates from green vegetation and roots, plays important roles in the production of polysaccharides, mannitol, vitamins‐K, bacteriocins, and the hydrolysis of α‐galactosides (Hemme & Foucaud‐Scheunemann, 2004; Sybesma et al., 2003). In all, Firmicutes and *Leuconostoc* may have crucial importance in the digestion of plant and other plant wall components (Escobar‐Correas et al., 2019).

Proteobacteria is related to environmental adaptation due to its ability to secrete lipase, protease, and amylase (Pemberton et al., 1997). *Aeromonas* within Proteobacteria has been identified as dominant and indigenous microbiota of silver carp (*Hypophthalmichthys molitrix*) that aids in digestion (Khurana et al., 2021). Previous studies have also demonstrated that Proteobacteria mainly associated with energy accumulation in mammals (Amato et al., 2014; Chevalier et al., 2015). This phylum may help *P. canaliculata* to digest food and accumulate energy, further adapting seasonal variations (Kaakoush, 2015).

### 4.2 Seasonal variation of dominant microbiome in the intestinal microbes of *P. canaliculata*

Bacteroidetes, Gammaproteobacteria, Enterobacteriaceae, Chloroflexaceae, *Aeromonas*, *Shewanella*, *Acinetobacter*, *Citrobacter*, and *Klebsiella* were enriched in summer (Figure 4b). Bacteroidetes plays a critical role in the degradation of carbohydrates and promotes the development of the gastrointestinal immune system (Jami et al., 2013). Previously study reported that Bacteroidetes was highly rich in *P. canaliculata*, indicating that Bacteroidetes can degrade high molecular weight organic matter (Zhou et al., 2022). *P. canaliculata* feeds on various abundant diet resources in summer. By contrast, the food resource of *P. canaliculata* is scarce due to cold temperature in winter. The function of this phylum was related to utilize the diet resource in summer for animal growth and reproduction and maintain immune homeostasis in higher temperature.

Actinobacteria, Bacillaceae, Nostocaceae, and *Bacillus* were enriched in autumn (Figure 4b). Actinobacteria as gram‐positive bacteria distributed in the terrestrial or aquatic environment (Servin et al., 2008). Actinobacteria was thought to be a dominant glucose degrader and was pivotal in the maintenance of gut homeostasis (Binda et al., 2018; Ito et al., 2012). Actinobacteria enriched in the intestine microbiome of *Litopenaeus vannamei*, which as gram‐positive and gram‐negative (Proteobacteria), were kept in balance in order to maintain the organic homeostasis (Fan et al., 2019). Moreover, Actinobacteria were well‐known bioactive natural product producers (van Keulen & Dyson, 2014), which can be used to isolate the potential probiotics (Bernal et al., 2015). It was speculated that Actinobacteria in the gut microbiota of *P. canaliculata* maintains the gut homeostasis.

**FIGURE 6:**
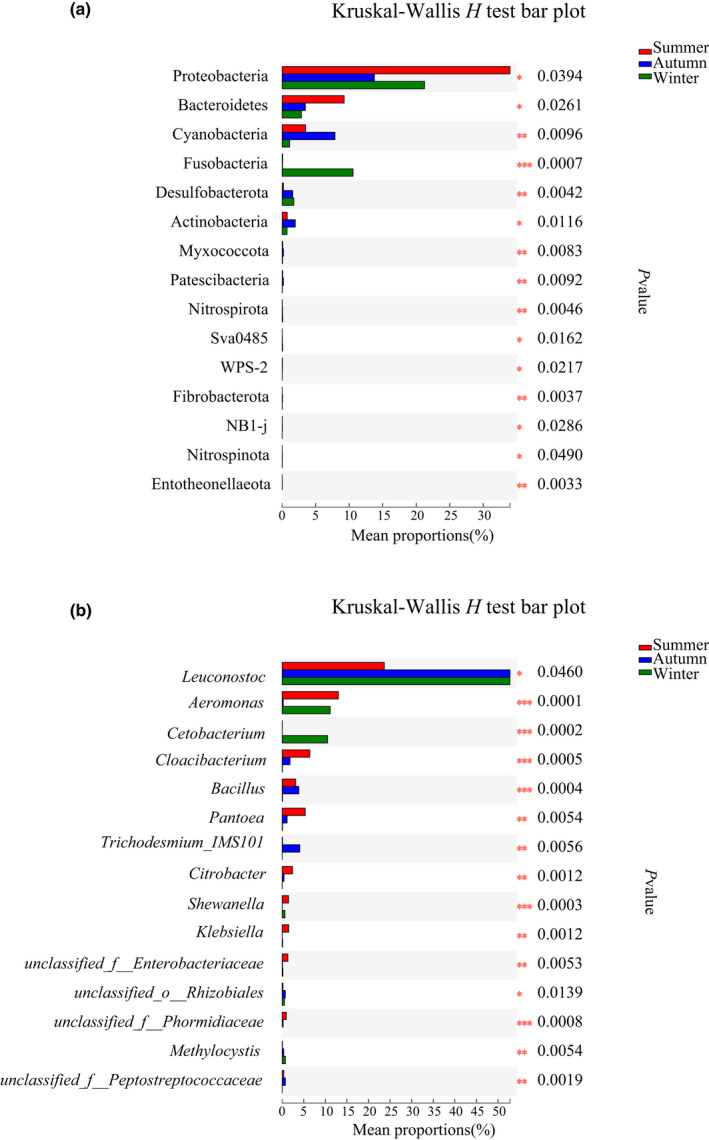
(a): Comparison of gut microbiota composition abundances of *P. canaliculata* at the phylum level in different seasons; (b): comparison of gut microbiota composition abundances of *P. canaliculata* at the genus level in different seasons.

The relative abundance of Cyanobacteria was significantly higher in autumn than those in other two groups (Figure 6a). The typical seasonal dynamics of phytoplankton in ecosystems usually consist of two peaks: winter–spring and autumn. Algae autumnal blooms may be the primary biological factors that cause changes in the proportion of Cyanobacteria (Xie et al., 2021). A high proportion of Cyanobacteria in the guts of invasive silver carp (*Hypophthalmichthys molitrix*), indicating a connection to their green algae feeding habits in the Mississippi River Basin (Ye et al., 2014). Dietary items of *P. canaliculata* in stomach contents included amorphous detritus, macrophytes, cyanobacteria, diatoms, green algae, and invertebrate parts (Kwong et al., 2010). The high abundance of Cyanobacteria was probably associated with the green algae in dietary of *P. canaliculata*.

The relative abundance of *Bacillus* and Bacillaceae was higher in the summer and autumn groups than in the winter group (Figures 4b and 6b). *Bacillus* and Bacillaceae were important cellulolytic bacteria in Firmicutes. Cellulose comprised the main ingredients of plant, which was the primary food source. *Bacillus* from Bacillaceae had unique properties on their hosts, such as inhibition of pathogenic bacteria, accelerated growth, and increased immunity. They also produce various extracellular enzymes such as amylase, protease, lipase, further aiding in digestion (Ray et al., 2012). The reason for their higher abundance in summer and autumn may be due to the abundant kinds of food in summer and autumn comparing with winter, and this microbiome may be involved in plant degradation.

The relative abundance of beneficial bacteria such as *Fusobacteria* and *Cetobacterium* increased significantly in winter to maintain the energy supply and immune system homeostasis of *P. canaliculata* in a cold environment (Figure 6a,b). The relative abundance of Fusobacteria in winter was at rather high levels, contrary to summer and autumn. *Fusobacteria* are anaerobic, Gram‐negative bailli (Bennett & Eley, 1993), associated with a protein‐rich diet, which exhibit proteolytic activity (Soverini et al., 2016). It digests carbohydrates into short‐chain fatty acids and butyrate. Butyrate provides many benefits to the host, including providing a majority of the energy supply to gastrointestinal cells (Collinder et al., 2003; von Engelhardt et al., 1998). Moreover, short‐chain fatty acids levels can directly affect substrate and energy metabolism, including skeletal muscle and liver (Larsen et al., 2014; Rimoldi et al., 2018). Evidence has shown that *P. canaliculata* could enhance their cold hardiness by energy accumulation (Matsukura & Wada, 2007; Matsukura et al., 2009). Significant enrichment for *Fusobacteria* in winter could be explained by the more reservation of heat energy and the heat consumption of *P. canaliculata* in winter.

Genus *Cetobacterium* within the phylum Fusobacteria were more abundant in winter comparing with other two groups (Figure 6b). This bacterium produced vitamin B_12_ at high efficiency and was capable of glucose fermentation (Tsuchiya et al., 2008). *Cetobacterium* as a common anaerobic inhabitats of the channel catfish (*Ictalurus punctatus*) is involved in vitamin metabolism and the production of antimicrobial peptides, which suggests the beneficial effects of this bacterium for its host (Bledsoe et al., 2018). The presence of *Cetobacterium* in *P. canaliculata* is probably beneficial for the host.

### 4.3 Seasonal variation of predicted function and function pathways in the intestinal microbes of *P. canaliculata*

In this study, metabolism, genetic information processing, environmental information processing, human diseases, cellular processes, and organismal systems play important roles in the adaptation to abundant food resources, consistent with the results previously reported in *P. canaliculata* among female group, male group, and juvenile group (Chen et al., 2021). Our results indicated that no difference was detected in the functional analysis of gut microbiota in *P. canaliculata*. Metagenomic approach can be used to illustrate the interactions of microbial structure and function in *P. canaliculata* in further studies.

## 5 CONCLUSION

In this study, significant differences were found in the diversity and structure of the intestinal microbiota of *P. canaliculata* among different seasons. The diversity of intestinal flora of *P. canaliculata* was the highest in summer and the lowest in winter. The results indicated that variations in food abundance caused by seasonal change have an impact on the intestinal microbiota of *P. canaliculata*. This study will provide insights into understanding the adaptive strategies of *P. canaliculata* to environmental changes. Further study will focus on the interaction between the gut microbiota and the host.

## AUTHOR CONTRIBUTIONS

**Shuxian Li:** Formal analysis (equal); methodology (equal); software (equal); writing – original draft (equal). **Zijin Qian:** Data curation (equal); formal analysis (equal); writing – original draft (equal). **Jiani Yang:** Writing – review and editing (equal). **Youfu Lin:** Writing – review and editing (equal). **Lian Chen:** Conceptualization (lead); project administration (lead); supervision (lead); writing – review and editing (lead).

## ACKNOWLEDGEMENTS

We thank Yanfu Qu for assistance in data analysis.

## FUNDING INFORMATION

This work was supported by the National Natural Science Foundation of China (No. 32170434), Postgraduate Research & Practice Innovation Program of Jiangsu Province (No. KYCX22_1613), Province and the Priority Academic Program Development of Jiangsu Higher Education Institutions (PAPD).

## CONFLICT OF INTEREST

The authors declare no conflicts of interest.

## Supporting information


Figure S1

Table S1‐S4

## Data Availability

The raw data are available from the SRA database (PRJNA834616).
