# Supplementary figures and images for: Seasonal variation in structure and function of gut microbiota in Pomacea canaliculata
Source: Ecol Evol. 2022 Jul 29;12(8):e9162. doi: 10.1002/ece3.9162 (PMC9336170; doi:10.1002/ece3.9162)

# Rarefaction curves

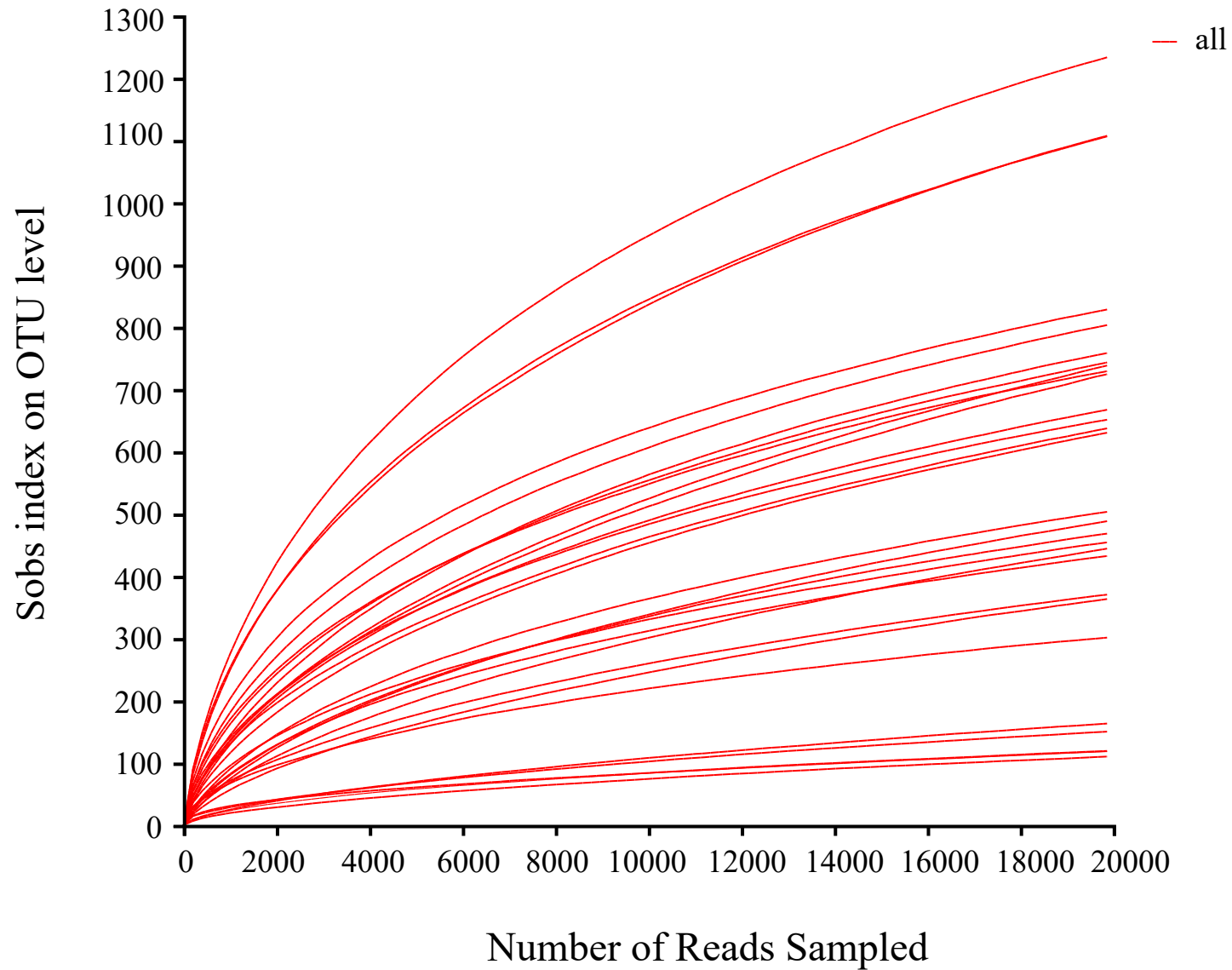

Supplement: Supplementary file 1 — Figure S1 [file ECE3-12-e9162-s002.pdf]
