# Supplementary material for: Seasonal variation in structure and function of gut microbiota in Pomacea canaliculata
Source: Ecol Evol. 2022 Jul 29;12(8):e9162. doi: 10.1002/ece3.9162 (PMC9336170; doi:10.1002/ece3.9162)
Supplement: Supplementary file 2 — Table S1‐S4 [file ECE3-12-e9162-s001.docx]

| Group | Shannon index | Simpson index | Chao index | Ace index |
| --- | --- | --- | --- | --- |
| Summer | 2.7800 | 0.2300 | 853.7400 | 1031.9000 |
| Autumn | 2.5900 | 0.3500 | 1123.2000 | 1365.2000 |
| Winter | 1.8600 | 0.4800 | 581.2000 | 747.6900 |
| *P* value (Autumn-Summer) | 0.6985 | 0.2453 | 0.0845 | 0.0221 |
| *P* value (Autumn-Winter) | 0.2225 | 0.0661 | 0.0218 | 0.0281 |
| *P* value (Summer-Winter) | 0.2048 | 0.1743 | 0.2390 | 0.2390 |

TABLE S1. Alpha diversity index of gut microbiota of *Pomacea canaliculata*

TABLE S2. The LEfSe analysis of gut microbiota composition of *Pomacea canaliculata* in each group (|LDA score| > 3.5, *P*<0.05).

| Taxa | Group | LDA value | *P* value |
| --- | --- | --- | --- |
| c_Gammaproteobacteria | Summer | 5.0518 | 0.0032 |
| p_Proteobacteria | Summer | 4.9869 | 0.0394 |
| f_Aeromonadaceae | Summer | 4.7921 | 0.0001 |
| o_Aeromonadales | Summer | 4.7921 | 0.0001 |
| *g_Aeromonas* | Summer | 4.7910 | 0.0001 |
| o_Enterobacterales | Summer | 4.6768 | 0.0067 |
| c_Bacteroidia | Summer | 4.4840 | 0.0254 |
| p_Bacteroidetes | Summer | 4.4814 | 0.0273 |
| f_Erwiniaceae | Summer | 4.4057 | 0.0059 |
| *g_Pantoea* | Summer | 4.4056 | 0.0059 |
| f_Enterobacteriaceae | Summer | 4.3466 | 0.0028 |
| *g_Citrobacter* | Summer | 3.9816 | 0.0012 |
| *g_Klebsiella* | Summer | 3.8357 | 0.0010 |
| *g_Shewanella* | Summer | 3.8115 | 0.0002 |
| f_Shewanellaceae | Summer | 3.8115 | 0.0002 |
| o_Alteromonadales | Summer | 3.8115 | 0.0002 |
| *g_unclassified_f_Enterobacteriaceae* | Summer | 3.8084 | 0.0053 |
| f_Chloroflexaceae | Summer | 3.7963 | 0.0047 |
| o_Chloroflexales | Summer | 3.7955 | 0.0099 |
| o_Bacteroidales | Summer | 3.7610 | 0.0495 |
| *g_unclassified_f_Phormidiaceae* | Summer | 3.7163 | 0.0008 |
| *g_unclassified_f_Chloroflexaceae* | Summer | 3.6329 | 0.0015 |
| f_Moraxellaceae | Summer | 3.6205 | 0.0015 |
| *g_Acinetobacter* | Summer | 3.6205 | 0.0015 |
| f_unclassified_o_Oxyphotobacteria_Incertae_Sedis | Summer | 3.5155 | 0.0084 |
| o_Oxyphotobacteria_Incertae_Sedis | Summer | 3.5155 | 0.0084 |
| *g_Leuconostoc* | Autumn | 5.1540 | 0.0460 |
| o_Cyanobacteriales | Autumn | 4.5467 | 0.0033 |
| *g_Trichodesmium_IMS101* | Autumn | 4.3943 | 0.0052 |
| o_Bacillales | Autumn | 4.3158 | 0.0005 |
| f_Bacillaceae | Autumn | 4.3103 | 0.0005 |
| *g_Bacillus* | Autumn | 4.2776 | 0.0004 |
| p_Actinobacteria | Autumn | 3.7388 | 0.0115 |
| f_Nostocaceae | Autumn | 3.7258 | 0.0036 |
| f_Beijerinckiaceae | Autumn | 3.6619 | 0.0338 |
| c_Actinobacteria | Autumn | 3.6092 | 0.0089 |
| p_Desulfobacterota | Winter | 4.0280 | 0.0042 |
| *g_Methylocystis* | Winter | 3.6641 | 0.0050 |
| c_Desulfobacteria | Winter | 3.6303 | 0.0003 |
| o_Desulfobacterales | Winter | 3.6169 | 0.0003 |
| f_Desulfosarcinaceae | Winter | 3.6150 | 0.0003 |
| *g_Sva0081_sediment_group* | Winter | 3.5916 | 0.0003 |

The first letter c, g, f, o, p in Taxa represent class, genus, family, order, phylum, respectively.

TABLE S3. The comparison of gut microbiota composition abundances of *P. canaliculata* among autumn, summer, and winter at phylum level through Kruskal-Wallis *H* test.

| Phyla | Relative abundance (%) | | | *H* | *P* value |
| --- | --- | --- | --- | --- | --- |
|  | Autumn | Summer | Winter |  |  |
| Proteobacteria | 13.7600 | 33.9900 | 21.2400 | 6.4664 | 0.0394 |
| Bacteroidetes | 3.4570 | 9.2450 | 2.8680 | 7.2951 | 0.0261 |
| Cyanobacteria | 7.8740 | 3.4860 | 1.1180 | 9.2970 | 0.0096 |
| Fusobacteria | 0.0468 | 0.0664 | 10.5700 | 14.6463 | 0.0007 |
| Desulfobacterota | 1.5680 | 0.1973 | 1.7360 | 10.9392 | 0.0042 |
| Actinobacteria | 1.9540 | 0.7470 | 0.7179 | 8.9218 | 0.0116 |
| Myxococcota | 0.1939 | 0.0751 | 0.0468 | 9.5736 | 0.0083 |
| Patescibacteria | 0.1798 | 0.0645 | 0.0201 | 9.3743 | 0.0092 |
| Nitrospirota | 0.0473 | 0.0064 | 0.0705 | 10.7846 | 0.0046 |
| Sva0485 | 0.0206 | 0.0037 | 0.0590 | 8.2464 | 0.0162 |
| WPS-2 | 0.0272 | 0.0316 | 0.0022 | 7.6580 | 0.0217 |
| Fibrobacterota | 0.0413 | 0.0009 | 0.0115 | 11.1771 | 0.0037 |
| NB1-j | 0.0237 | 0.0060 | 0.0043 | 7.1088 | 0.0286 |
| Nitrospinota | 0.0196 | 0.0009 | 0.0079 | 6.0304 | 0.0490 |
| Entotheonellaeota | 0.0060 | 0.0014 | 0.0000 | 11.4259 | 0.0033 |

TABLE S4. The comparison of gut microbiota composition abundances of *P. canaliculata* among autumn, summer, and winter at genus level through Kruskal-Wallis *H* test.

| Genera | Relative abundance (%) | | | *H* | *P* value |
| --- | --- | --- | --- | --- | --- |
|  | Autumn | Summer | Winter |  |  |
| *Leuconostoc* | 52.7800 | 23.6600 | 52.7500 | 6.1599 | 0.0460 |
| *Aeromonas* | 0.2407 | 13.0200 | 11.1200 | 18.4528 | 0.0001 |
| *Cetobacterium* | 0.0030 | 0.0133 | 10.5200 | 17.5221 | 0.0002 |
| *Cloacibacterium* | 1.8220 | 6.3980 | 0.0540 | 15.3263 | 0.0005 |
| *Bacillus* | 3.8470 | 3.1400 | 0.0662 | 15.4889 | 0.0004 |
| *Pantoea* | 1.1390 | 5.3540 | 0.0726 | 10.4590 | 0.0054 |
| *Trichodesmium_IMS101* | 4.0850 | 0.0728 | 0.0007 | 10.3696 | 0.0056 |
| *Citrobacter* | 0.4240 | 2.3950 | 0.0252 | 13.3806 | 0.0012 |
| *Shewanella* | 0.0106 | 1.4830 | 0.6337 | 16.3082 | 0.0003 |
| *Klebsiella* | 0.0146 | 1.5210 | 0.1043 | 13.4480 | 0.0012 |
| *unclassified_f__Enterobacteriaceae* | 0.0695 | 1.3180 | 0.1547 | 10.4926 | 0.0053 |
| *unclassified_o__Rhizobiales* | 0.7241 | 0.1712 | 0.5229 | 8.5589 | 0.0139 |
| *unclassified_f__Phormidiaceae* | 0.2210 | 0.9663 | 0.0000 | 14.3642 | 0.0008 |
| *Methylocystis* | 0.3625 | 0.0508 | 0.7445 | 10.4285 | 0.0054 |
| *unclassified_f__Peptostreptococcaceae* | 0.7427 | 0.3667 | 0.0029 | 12.5795 | 0.0019 |
